# Deciphering the adsorption machinery of Deep-Blue and Vp4, two myophages targeting members of the *Bacillus cereus* group

**DOI:** 10.1128/jvi.00745-24

**Published:** 2024-08-23

**Authors:** Manon Nuytten, Audrey Leprince, Adeline Goulet, Jacques Mahillon

**Affiliations:** 1 Laboratory of Food and Environmental Microbiology, Earth and Life Institute, Université Catholique de Louvain (UCLouvain), Louvain-la-Neuve, Belgium; 2 Laboratoire d’Ingénierie des Systèmes Macromoléculaires (LISM), Institut de Microbiologie, Bioénergies et Biotechnologie (IM2B), CNRS and Aix-Marseille Université UMR7255, Marseille, France; Michigan State University, East Lansing, Michigan, USA

**Keywords:** *Bacillus cereus*, carbohydrate-binding module, ectolysin, myophages, receptor-binding protein

## Abstract

In tailed phages, the baseplate is the macromolecular structure located at the tail distal part, which is directly implicated in host recognition and cell wall penetration. In myophages (i.e., with contractile tails), the baseplate is complex and comprises a central puncturing device and baseplate wedges connecting the hub to the receptor-binding proteins (RBPs). In this work, we investigated the structures and functions of adsorption-associated tail proteins of Deep-Blue and Vp4, two *Herelleviridae* phages infecting members of the *Bacillus cereus* group. Their interest resides in their different host spectrum despite a high degree of similarity. Analysis of their tail module revealed that the gene order is similar to that of the *Listeria* phage A511. Among their tail proteins, Gp185 (Deep-Blue) and Gp112 (Vp4) had no structural homolog, but the C-terminal variable parts of these proteins were able to bind *B. cereus* strains, confirming their implication in the phage adsorption. Interestingly, Vp4 and Deep-Blue adsorption to their hosts was also shown to require polysaccharides, which are likely to be bound by the arsenal of carbohydrate-binding modules (CBMs) of these phages’ baseplates, suggesting that the adsorption does not rely solely on the RBPs. In particular, the BW Gp119 (Vp4), harboring a CBM fold, was shown to effectively bind to bacterial cells. Finally, we also showed that the putative baseplate hub proteins (i.e., Deep-Blue Gp189 and Vp4 Gp110) have a bacteriolytic activity against *B. cereus* strains, which supports their role as ectolysins locally degrading the peptidoglycan to facilitate genome injection.

**IMPORTANCE:**

The *Bacillus cereus* group comprises closely related species, including some with pathogenic potential (e.g., *Bacillus anthracis* and *Bacillus cytotoxicus*). Their toxins represent the most frequently reported cause of food poisoning outbreaks at the European level. Bacteriophage research is undergoing a remarkable renaissance for its potential in the biocontrol and detection of such pathogens. As the primary site of phage-bacteria interactions and a prerequisite for successful phage infection, adsorption is a crucial process that needs further investigation. The current knowledge about *B. cereus* phage adsorption is currently limited to siphoviruses and tectiviruses. Here, we present the first insights into the adsorption process of *Herelleviridae* Vp4 and Deep-Blue myophages preying on *B. cereus* hosts, highlighting the importance of polysaccharide moieties in this process and confirming the binding to the host surface of Deep-Blue Gp185 and Vp4 Gp112 receptor-binding proteins and Gp119 baseplate wedge.

## INTRODUCTION

The *Bacillus cereus* group, or *B. cereus sensu lato* (*s.l*.), is a genetically related cluster of Gram-positive, rod-shaped, and spore-forming bacteria (1). The group taxonomy is highly debated, and more than 30 species are now recognized as members, including species with different lifestyles and ecology (2, 3). Among them, some strains of *B. cereus sensu stricto* (*s.s*.), *Bacillus weihenstephanensis* and *Bacillus cytotoxicus*, are known to be involved in foodborne intoxication and infection (4–6).

Bacteriophages (phages) are viruses that specifically infect and kill bacteria or archaea. After decades as primary models for the study of fundamental biological processes, phages are experiencing renewed interest in the food industry, particularly for the detection and biocontrol of foodborne pathogens (7, 8). The majority of phages documented to date fall within the *Caudoviricetes* class, encompassing those characterized by a tail attached to their capsid (9, 10). The tail is the primary structure involved in the early stages of the viral life cycle consisting in the phage adsorption onto its host, followed by genome injection (11–13). The adsorption is highly dependent on the specific interaction between receptors on the bacterial surface, on the one hand, and viral proteins called receptor-binding proteins (RBPs), on the other (14). Receptors typically involve lipopolysaccharides and outer membrane proteins in Gram-negative hosts and teichoic acids or other secondary cell wall polysaccharides in Gram-positive bacteria, but other cell envelope components [e.g., capsular polysaccharides, peptidoglycan (PG), or surface proteins] and appendages (e.g., flagella and pili) have also been identified as receptors (15–19). On the phage side, the tail distal end forms a macromolecular structure called the baseplate, which attaches tail appendages involved in host recognition (20–22). In addition, some of these proteins can display enzymatic activities that facilitate the access to the receptor by degrading polysaccharides (i.e., depolymerase) or helping genome injection by breaking down the peptidoglycan meshwork (i.e., tail lysin/ectolysin) (23–26).

Overall, the baseplate structure greatly depends on the phage morphology, the nature of the receptor, and the bacterial hosts. The typical myophage baseplate is rather complex and is composed of a central hub, a cell-puncturing device involved in cell wall and membrane penetration, and baseplate wedges (BW), including at least three conserved structural proteins responsible for the attachment of the tail fibers or RBP through their N-extremity. RBPs often assemble into trimeric structures, which usually rely on chaperone activity (14). The binding of the RBPs to their bacterial receptors is accompanied by sheath contraction and baseplate conformational changes, resulting in the injection of the phage genetic material into the host (27). The simplest myophage baseplates are composed of six types of proteins, which are conserved among most contractile tail systems (e.g., myophage, type VI secretion system, and R-type pyocins) (28). Alternatively, the baseplate of *Escherichia coli* phage T4 consists of at least 15 different proteins and anchors two different types of tail fibers (i.e., short and long tail fibers) involved in different stages of the adsorption process (29–31). Concerning the myophages infecting Gram-positive bacteria, little information is yet available, and only the structure of the *Listeria* phage A511 has recently been investigated (32).

Recently, the DeepMind’s machine-learning protein structure prediction program AlphaFold2 (AF2) (33) has been proven to be extremely valuable in obtaining structures of phage tail proteins and of their complexes, as exemplified by the baseplate structure predictions of a few siphophages (34–37).

Here, we study the proteins assembling the baseplate of two myophages, Deep-Blue and Vp4, infecting members of the *B. cereus* group (38, 39). Despite their classification within the *Herelleviridae* family, bioinformatic analyses revealed disparities in their tail proteins, implying a divergence in their baseplate architecture compared to that of the prototypical phage A511. Among the tail proteins (TPs), Deep-Blue Gp185 and Vp4 Gp112 emerged as primary candidates involved in adsorption, as they lack homologs in *Listeria* A511 phage and exhibit less conservation at their C-terminal extremity, while their AF2 structure prediction suggests a trimeric structure typical of RBPs. Experimental approaches confirmed that both proteins bind to *B. cereus* cells, highlighting their role in phage adsorption. AF2 analysis also revealed multiple carbohydrate-binding modules (CBMs) within Deep-Blue and Vp4 TPs, likely facilitating the adsorption process, including Vp4 BW Gp119, whose binding capacity was confirmed.

## RESULTS

### The overall organization of Deep-Blue and Vp4 tail regions is similar to that of phage A511

**Fig 1:**
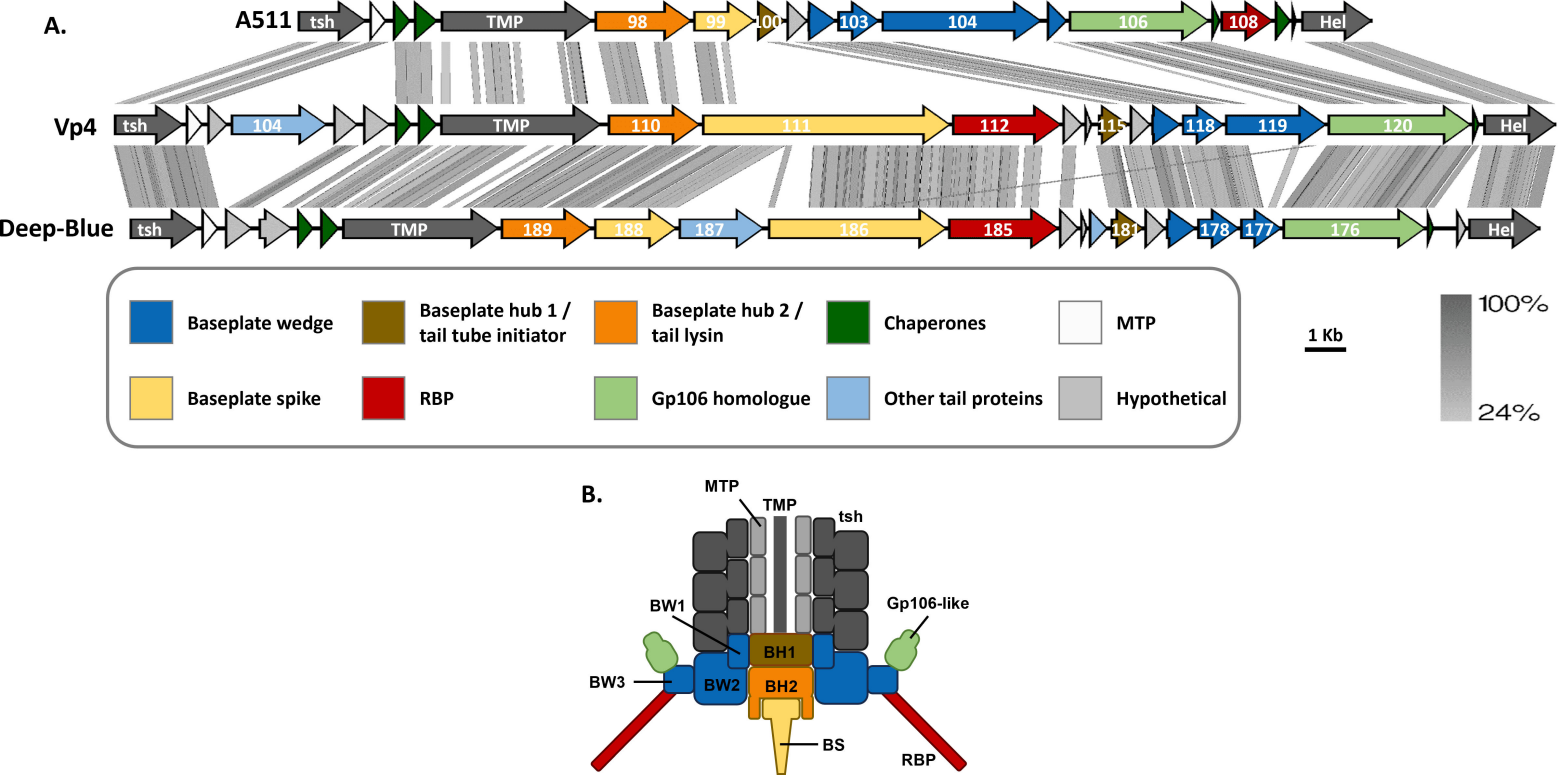
Tail proteins encoded by the myoviruses A511, Vp4, and Deep-Blue. (A) Alignment of the tail protein-encoding region of *Listeria* phage A511 and *B. cereus* phages Deep-Blue and Vp4. The gene comparison was generated with EasyFig using tBLASTx, and the gradient (gray) scale of gene identity is indicated on the right, together with the scale of the genetic loci (in kilobase). (B) Schematic representation of the simplest contractile tail and baseplate based on that of *E. coli* phage Mu (28) and *Listeria* phage A511 (32). The color coding corresponds to the one of part A. BW1, BW2, and BW3 correspond to Gp117/Gp179, Gp118/Gp178, and Gp119/Gp177, respectively, in phages Vp4/Deep-Blue. BH, baseplate hub; BS, baseplate spike; BW, baseplate wedge; Hel, helicase; MTP, major tail tube protein; RBP, receptor-binding protein; TMP, tape measure protein; tsh, tail sheath.

It was previously shown that, in several myophages of the *Herelleviridae* family, baseplate proteins are encoded in a region delimited by the tape measure protein (TMP) and helicase genes (40). Both Deep-Blue and Vp4 exhibit a similar organization, with well-conserved gene order (Fig. 1; Table S1).

The structures of the TPs of both phages were predicted using AF2 and submitted to the Dali server to retrieve structural homologs. The most significant hits are summarized in Table 1. Deep-Blue encodes unique proteins such as a hypothetical protein (Gp174), a putative cysteine peptidase (Gp182), and Gp187, an unknown TP absent in the Vp4 tail region. Interestingly, Vp4 encodes a putative depolymerase whose gene (*gp104*) is located upstream of the *tmp*. Deep-Blue and Vp4 TP gene order remains largely conserved compared to *Listeria* phage A511, with the only distinction being the location of the genes encoding the putative RBPs (Fig. 1A; Table S1). Table S2 highlights the structural homologs of Vp4 and Deep-Blue found in Gram-negative contractile tail systems, including *E. coli* phage tails, *Pseudomonas aeruginosa* R-type pyocin (41), the contractile injection system of *Serratia entomophila* (42), and the virulence cassette of *Photorhabdus* sp. (43).

**TABLE 1:** Relevant hits from AlphaFold2 predicted structures of Deep-Blue and Vp4 tail proteins submitted to the Dali server to retrieve related structures

	Deep-Blue			Vp4		
Tail proteins	Gp^*e*^ (size in aa)	Domain boundaries	Dali hit (PDB ID^*c*^, *Z*-score)	Gp^*e*^ (size in aa)	Domain boundaries	Dali hit (PDB ID^*c*^, *Z*-score)
Tail depolymerase	−^*f*^	−	−	104 (790)	92–605	GH87 alpha-1,3-glucanase (7C7D; 35.4)^*a*^
Baseplate hub 2/tail lysin	189 (746)	9–323	Mu Gp44 central hub (1WRU, 19.0)	110 (746)	9–316	Mu Gp44 central hub (1WRU, 19.9)
		450–534	Mu Gp44 central hub (1WRU, 19.0)		488–546	Mu Gp44 central hub (1WRU, 19.9)
		613–744	Spike of extracellular contractile injection system (6JOM, 18.0)^*a*^		651–744	Structure of VgrG1 in the type VI secretion (6H3N, 19.2)^*a*^
Central spike/needle	188 (679)	28–357	R2 pyocin membrane-piercing spike (4S37, 9.3)^*a*^	111 (2,033)	22–306	R2 pyocin membrane-piercing spike (4S37, 9.0)^*a*^
Central spike/needle	186 (1,463)	65–151 261–328	Xyloglucan-binding module (4BJ0, 13.8)^*b*^		740–889	Xylan-binding domain (1DYO, 11.9)^*b*^
		381–482	N-terminal endo-1,4-β-D-xylanase 10B (2W5F, 11.7)^*a*^		1116–1252	Tandem CBM domain of Xyn10C (4XUP, 13.7)^*b*^
		545–684	Tandem CBM domain of Xyn10C (4XUP, 12.3)^*b*^		1329–1483	Carbohydrate-binding domain of xylanase Xyn10B (1H6X, 14.6)^*b*^
		754–911	Xylan-binding domain from CBM22 (1DYO, 14.4)^*b*^		1806–1927	Endo-alpha-D-arabinanase (8IC1, 13.1)^*b*^
		916–1055	CBA120 Tail Spike 4 (5W6H, 15.2)			
		1208–1357	Family 16 CBM (2ZEY; 15.4)^*b*^			
Tail protein	187 (701)	282–586	phicrAss001 portal protein (7QOL, 8.7)	−	−	−
RBP	185 (906)	337–445	PKD domain of collagenase (2Y72, 9.3)	112 (895)	335–448	PKD domain of collagenase (4JRW, 8.0)
		637–867	T5 L-shaped tail fiber protein (carboxy terminal + chaperone) (4UW8, 5.8)		637–867	T5 L-shaped tail fiber protein (carboxy terminal + chaperone) (4UW8, 5.3)
Cysteine peptidase	182 (163)	2–163	Cysteine peptidase from *B. cereus* ATCC^*d*^ 10987 (3KW0, 15.1)	−	−	−
Baseplate hub 1	181 (275)	13–149	Antifeeding prophage Afp7 hub (6RBK, 9.8)	115 (257)	13–149	Antifeeding prophage Afp7 hub (6RBK, 10.0)
Baseplate wedge 1	179 (249)	No hit	No hit	117 (246)	No hit	No hit
Baseplate wedge 2	178 (348)	1–191	Antifeeding prophage Afp1 ring structure (6RAO, 9.1)	118 (348)	1–191	Antifeeding prophage Afp1 ring structure (6RAO, 13.2)
		192–348	T4 gp6 BW (3H3W, 10.0)		192–348	T4 gp6 BW (3H3W, 14.0)
		5–348	T4 gp7 BW (5H × 2, 14.3)		5–348	T4 gp7 BW (5H × 2, 13.2)
Baseplate wedge 3	177 (341)	192–339	A511 gp105 BW3 (6HHK, 19.4)	119 (831)	268–425	Family 16 carbohydrate-binding module (2ZEY, 15.4)^*b*^
					486–584	A511 gp105 BW3 (6HHK, 6.3)
					685–799	Carbohydrate-binding domain of xylanase Xyn10B (1H6X, 11.7)^*b*^
Tail fiber/VrlC	176 (1,169)	811–959	Lectin-like domain of Cwp84, surface layer-associated protein (4CI7, 8.5)^*b*^	120 (1,174)	814–963	Lectin-like domain of Cwp84, surface layer-associated protein (4CI7, 9.4)^*b*^
		1060–1164	Endo-beta-N-acetylglucosaminidase A (3FHA, 10.5)^*b*^		1065–1170	Family 16 carbohydrate-binding module 1 (2ZEW, 9.7)^*b*^
Chaperone	175 (63)	No hit	No hit	121 (64)	No hit	No hit

^*a*^ Indicates putative catalytic domain.

^*b*^ Indicates putative carbohydrate-binding module folds.

^*c*^ PDB ID, Protein Data Bank ID.

^*d*^ ATCC, American Type Culture Collection.

^*e*^ Gp, gene product.

^*f*^ −, protein not present in the phage.

### Vp4 Gp112 and Deep-Blue Gp185 display typical RBP features

**Fig 2:**
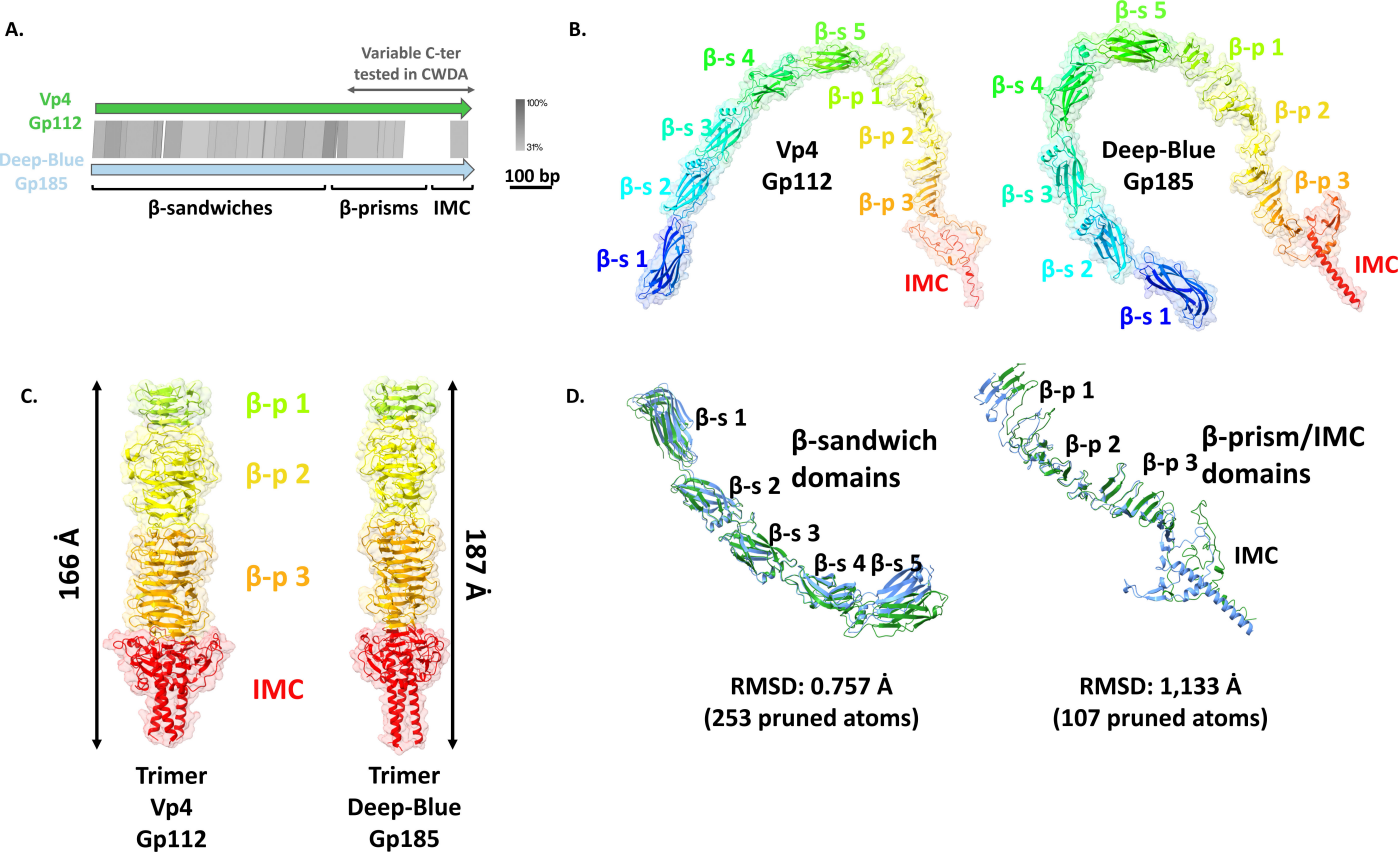
*In silico* analysis of Vp4 (Gp112) and Deep-Blue (Gp185) RBPs. (A) Alignment of Gp112 and Gp185 coding genes generated with EasyFig using tBLASTx. The gradient (gray) scale of gene identity and the scale of the genetic loci are indicated on the right. Domains identified by AlphaFold are indicated, together with the C-terminal part tested in CWDA. (B) Ribbon and transparent surface representation of the predicted three-dimensional structure of Gp112 (left) and Gp185 (right) monomer colored by rainbow N- to C-termini, comprising, from the N- to the C-terminal extremity, five structural β-sandwiches, stretches of β-strands that will assemble into β-prims, and the C-terminal intramolecular chaperone domain. (C) Ribbon and transparent surface representation of the trimeric β-prisms and C-terminal IMC domain of Gp112 (left) and Gp185 (right). (D) Superposition of the N-ter β-sandwiches of Gp112 (green) and Gp185 (blue) (left) and of the C-ter β-prism and IMC domains of Gp112 (green) and Gp185 (blue) (right). β-p, β-prism; β-s, β-sandwich; IMC, intramolecular chaperone; RMSD, root mean square deviation; CWDA, cell wall decoration assay.

Gp112 and Gp185 are the best RBP candidates for Vp4 and Deep-Blue, respectively. First, no similarity could be found between their genes and any of the A511 tail region, which is expected from tail proteins involved in the recognition of variable components at their host surface (Fig. 1A). Second, the C-terminal extremities of Gp185 and Gp112 are far less conserved, which could also account for the differences in host ranges between the two *Bacillus* phages (Fig. 2A). Notably, the RBP C-termini are also among the most variable regions when aligning TP modules of myophages infecting the *B. cereus* group (Fig. S1, deep-red rectangles). Additionally, the Gp185 and Gp112 predicted structures display five β-sandwich domains at their N-termini, followed by stretches of β-strands and a C-ter intramolecular chaperone (IMC) (Fig. 2B; Fig. S2). Likewise, the RBPs of streptococcal phages were previously shown to harbor four β-sandwiches at their N-termini (35). No CBM fold was identified in both candidate RBPs (Fig. 2B; Table 1). As most RBPs described to date, Gp112 and Gp185 were predicted to form trimers (14), even if their N-ter regions (i.e., the β-sandwich domains) could not be trimerized in our predictions (Fig. 2C; Fig S2). Those trimeric structures display three β-prisms (resulting from the assembly of the stretches of β-strands identified in the monomeric structure) and the C-terminal IMC. Finally, the predicted monomeric structures of Deep-Blue Gp185 and Vp4 Gp112 are highly similar across their entire length (Fig. 2D) with root mean square deviations (RMSDs, indicating the variability between the two structures) of 0.757 and 1.133 Å for the β-sandwich domains and β-prism/IMC domains, respectively.

### Deep-Blue and Vp4 candidate RBPs bind to *B. cereus s.l*. strains

The RBP candidates of Deep-Blue (Gp185) and Vp4 (Gp112) were fused to an N-terminal green fluorescent protein (GFP) to assess their host-binding function. Since the purification of the entire proteins fused to the GFP was unsuccessful, only their C-terminal variable parts (comprising β-prism 2, β-prism 3, and IMC domains) were fused to an N-terminal GFP and further used in cell wall decoration assays (Fig. 2A). Strains with different phage sensitivities were assayed, as host range not always correlates with the phage adsorption: sensitive strains, characterized by plaque formation in spotting assays; insensitive strains, no lysis plaque or lysis zone observed; and lysed strains, strains affected by lysis only at a high multiplicity of infection [i.e., resulting from the “lysis from without” phenomenon or abortive infection defense mechanisms (44, 45)]. As the latter does not lead to any production of phage progeny (i.e., non-productive infection), these strains were not considered host strains.

**Fig 3:**
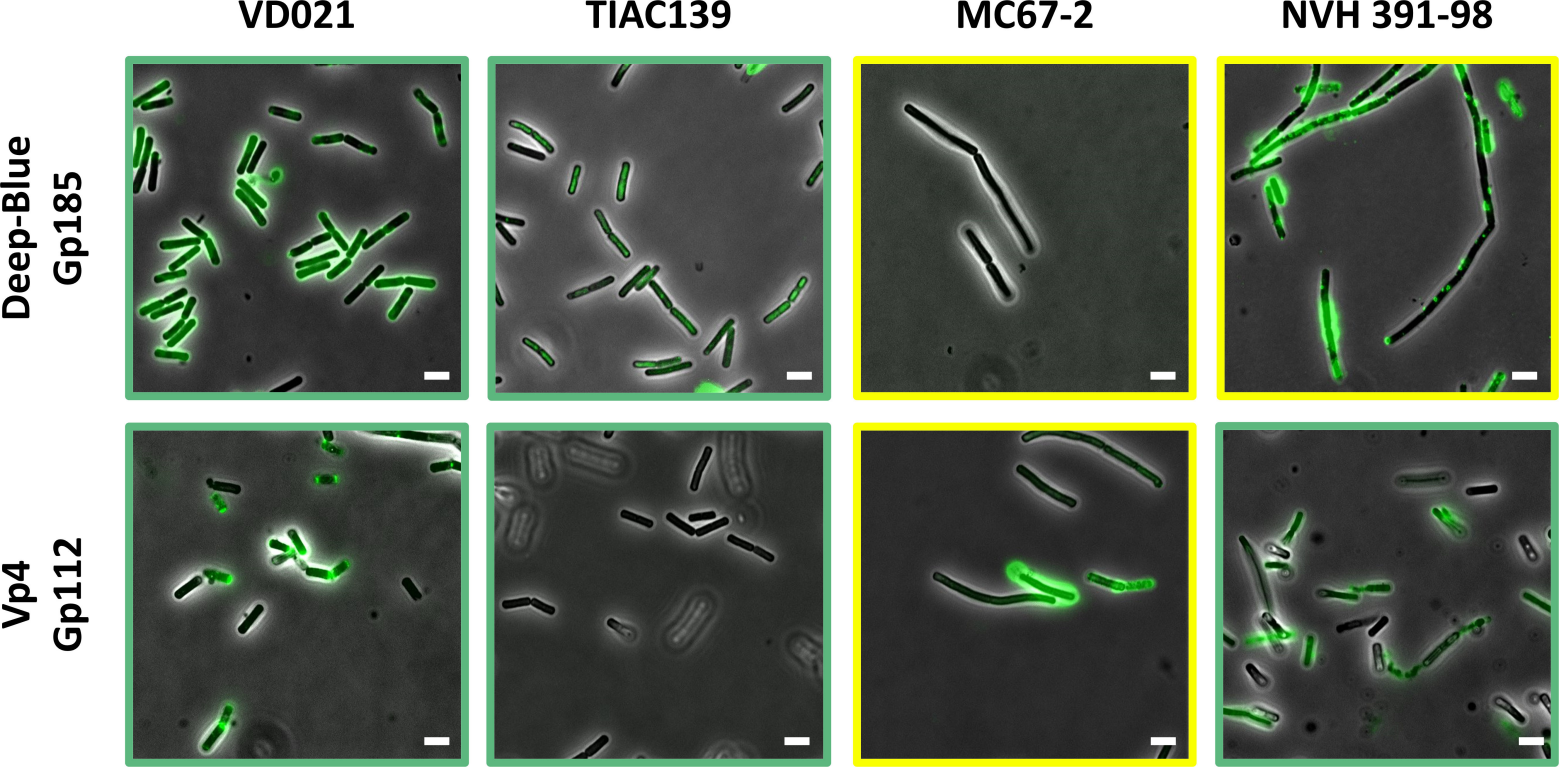
Cell wall decoration assay of Deep-Blue Gp185 and Vp4-Gp112 to bacteria of the *B. cereus* group. The C-terminal part of each candidate RBP was fused to a GFP tag to assess their binding abilities. Exponentially growing *Bacillus* cells were incubated with ca. 20–50 μg of proteins and observed using an epifluorescence microscope. The tested strains are indicated on the top. The overlays between the contrast phase and fluorescent images are shown. The scales represent 5 µm. Green frames represent sensitive strains, while yellow frames represent lysed strains.

Cell wall decoration assay results are illustrated in Fig. 3 and summarized in Table 2, together with the phage host ranges. Strains that are insensitive to phage infections (e.g., *B. cereus* ATCC 10987) were not decorated by Gp185 (Deep-Blue) or Gp112 (Vp4). Conversely, both proteins bound to the strains sensitive to their related phage, except for *B. cereus* TIAC139, not bound by Vp4 Gp112. Notably, both proteins were able to bind to most strains which are only affected by lysis, thereby exhibiting broader binding spectra than the host range of Deep-Blue and Vp4. The ability of Gp185 and Gp112 to bind to *B. cereus* strains confirmed their role as the RBP of Deep-Blue and Vp4, respectively. Interestingly, this observation of RBP-binding spectra broader than the host ranges of their parental phages could suggest the potential implication of other TPs in the adsorption processes of these *B. cereus s.l*. phages.

**TABLE 2:** Phages Deep-Blue and Vp4 host spectra and binding spectra of their putative RBPs^*e*^

	Deep-Blue		Vp4		
Strains	Gp185	Φ	Φ	Gp112	Reference
*B. cereus s.s.*					
ATCC 10987	−	IS	IS	−	(46)
F4810/72	+	L	L	+	(47)
H3081.97	+	L	L	+	(48)
TIAC139	+	S	S	−	Sciensano^*a*^
TIAC244	+	L	S	+	Sciensano
*B. cereus s.l.*					
VD021	+	S	S	+	(49)
*B. cytotoxicus*					
9SM2	+	L	S	+	MIAE^*b*^
E17.4	−	L	L	−	MIAE
E7.2	+	L	L	+	MIAE
NVH 391–98	+	L	S	+	INRAE^*c*^
PDT2.12	+	L	L	−	(50)
SM1.1	+	L	L	+	(50)
SM2.8	+	L	S	+	(50)
*Bacillus mycoides*					
KBS 2–12	+	L	S	+	MIAE
NRRL B-347	+	L	S	+	(51)
*Bacillus thuringiensis*					
DBT242	+	S	L	+	MIAE
GBJ001	+	L	S	+	(52)
HD73	+	S	S	+	BGSC^*d*^
T50001	+	S	L	+	MIAE
*B. weihenstephanensis*					
BtB2-4	+	S	L	+	(53)
KBAB4	−	L	L	+	(54)
LH002	−	IS	L	+	(55)
MC118-4	−	L	L	+	(56)
MC67-2	−	L	L	+	(56)
SI0170	−	L	IS	−	MIAE
Si0239	+	S	L	+	(39)
WS10202	+	S	L	+	(57)
WS10204	+	L	L	+	(57)

^*a*^ Sciensano, Belgian Institute for Health, Brussels, Belgium.

^*b*^ MIAE, Food and Environmental Lab, UCLouvain, Louvain-la-Neuve, Belgium.

^*c*^ INRAE, Institut National de Recherche pour l’Agriculture, l’Alimentation et l’Environnement, Jouy-en-Josas, France.

^*d*^ BGSC, *Bacillus* Genetic Stock Center, Ohio State University, Columbus, OH, USA.

^*e*^ The phage host spectra are shown in the “Φ” columns using the following codes: S, sensitive strains; L, strains affected by lysis; and IS, insensitive strains. The C-ter part of the putative RBP, Gp185 and Gp112, were fused to a GFP and tested on different strains of the *B. cereus* group, in a cell wall decoration assay. **+** and − indicate binding and no binding, respectively, of the corresponding proteins to the different strains.

### Deep-Blue and Vp4 encode various TPs displaying CBMs

**Fig 4:**
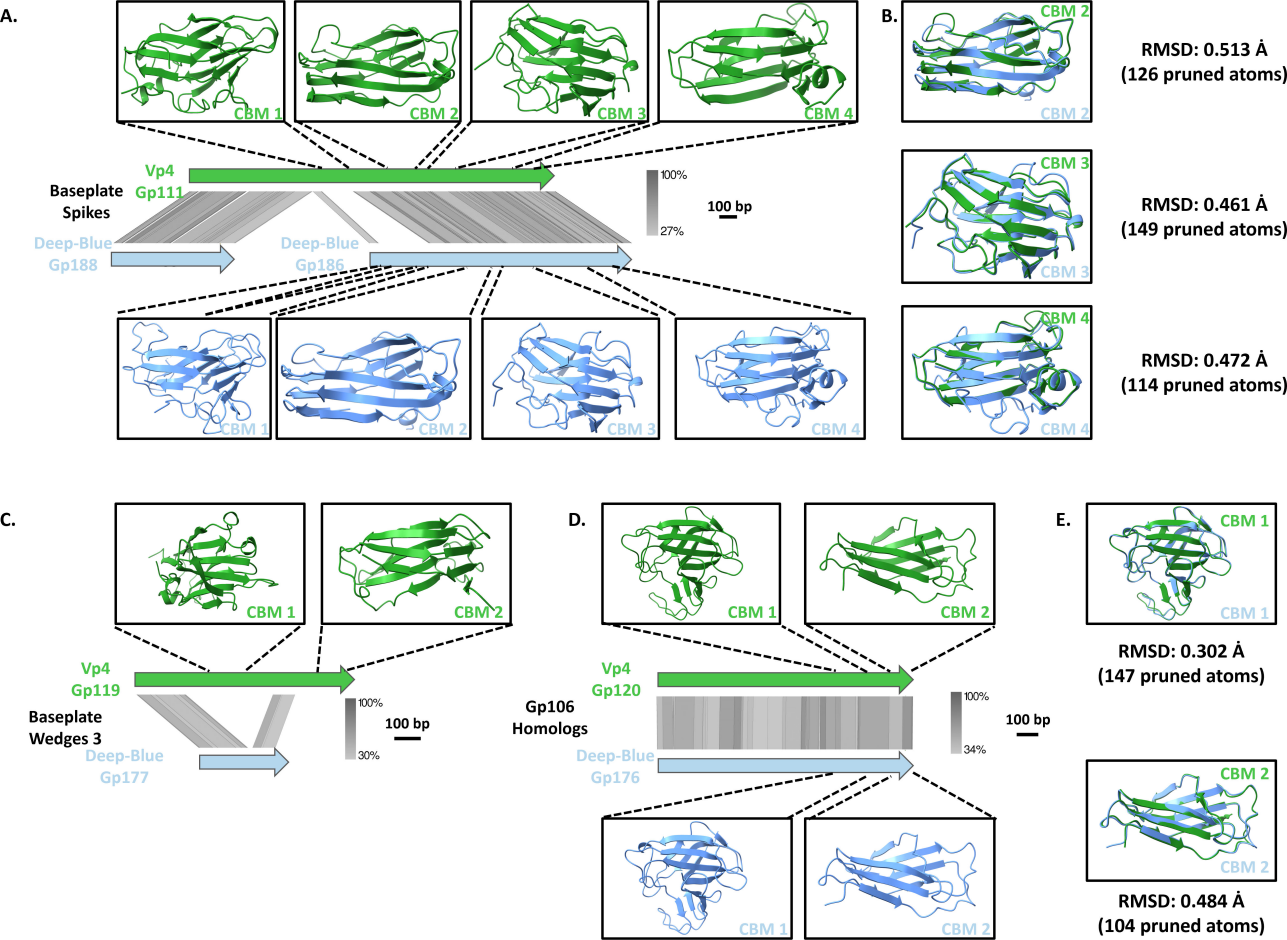
*In silico* analysis of Vp4 and Deep-Blue tail proteins displaying carbohydrate-binding modules. (A) Alignment of tail spike protein-coding regions of Vp4 Gp111 (green) and Deep-Blue Gp188 and Gp186 (blue). Ribbon representation of carbohydrate-binding modules (CBM) of Gp111 and Gp186. (B) Superposition of the CBM2, CBM3, and CBM4 of Gp111 (green) and Gp186 (blue). (C) Alignment of baseplate wedge 3 protein genes of Vp4 Gp119 (green) and Deep-Blue Gp177 (blue). The top panel shows the prediction of the Vp4 Gp119 CBM folds (green). (D) Alignment of A511 Gp106 homolog genes: Vp4 Gp120 (green) and Deep-Blue Gp176 (blue), with the predicted CBM folds of their respective proteins. (E) Superposition of the CBM1 (top) and CBM2 (bottom) predictions of Gp120 (green) and Gp176 (blue). All alignments were generated with EasyFig using tBLASTx. The gradient (gray) scale of gene identity and the scale of the genetic loci are indicated. The CBMs were retrieved from the Dali server, and their relative positions within their genes are represented with dotted lines.

Our AF2 predicted structures of all proteins assembling the Deep-Blue and Vp4 baseplates highlight that both phages contain multiple CBMs likely involved in the binding of polysaccharides upon host adsorption (Table 1; Fig. 4).

First, the tail spike protein of Vp4, Gp111, has two homologs in Deep-Blue: Gp188, which corresponds to the Gp111 N-terminal region (3–678 aa), and Gp186, which matches its C-terminal part (909–2,030 aa). While Gp188 and the N-terminus of Gp111 harbor structural and catalytic domains (Table 1), Deep-Blue Gp186 and Vp4 Gp111 C-terminus both contain four CBMs (Fig. 4A). Notably, only the three last CBMs (from CBM2 to CBM4) are conserved between the two proteins (Fig. 4B), while their CBM1 could not be structurally aligned with each other.

Second, in both phages, three proteins are putative components of the peripheric wedges surrounding the central hub and anchoring the tail fibers (Fig. 1B). While the first (Vp4-Gp117 and Blue-Gp179) and the second (Vp4-Gp118 and Blue-Gp178) BW proteins are conserved among contractile tail systems (Fig. 1A ; Table 1; Fig. S2), the third BW protein differs between Deep-Blue (Gp177) and Vp4 (Gp119). Indeed, only their N-terminal domains are conserved and homologous to the BW3 of A511 (i.e., Gp105), which forms the proximal part of the tail fibers and attaches the RBPs (i.e., Gp108 and Gp106) (Table S1). The predicted structures of this conserved domain in Deep-Blue and Vp4 superimpose well on the structure of their homologous domain in A511 Gp105 (RMSD_Gp105–Gp119_ = 1.028 Å and RMSD_Gp105–Gp177_ = 0.917 Å, data not shown). Interestingly, Vp4 Gp119 harbors two CBMs that are totally absent in its Deep-Blue (Gp177) and A511 (Gp105) homologs (Fig. 4C). This suggests that the BW3 of Vp4, Gp119, may not only serve to anchor the receptor-binding protein but could also actively contribute to host binding through carbohydrate interactions.

Finally, Deep-Blue Gp176 and Vp4 Gp120 are homologs of A511 Gp106 based on sequence alignment (45% and 43% protein sequence identity, respectively) (Fig. 1A). Gp106 homologs are known to be responsible for the double-layer aspect of the baseplate in some myophages and, in A511, Gp106 was shown to form pyramidal structures attached to the proximal part of the tail fiber, presumably interacting with the cell wall (CW) (32). The two CBMs located at the C-terminal end of Gp176 and Gp120 superimpose well onto each other (RMSD of 0.302 Å and 0.484 Å for CBM1 and CBM2, respectively), which highlights their structural homology (Fig. 4D and E). Interestingly, those CBMs are also conserved in A511-Gp106 (For the CBM1: RMSD_Gp106-Gp176_ = 0.418 Å and RMSD_Gp106-Gp120_ = 0.465 Å; For the CBM2: RMSD_Gp106-Gp176_ = 0.591 Å and RMSD_Gp106-Gp120_ = 0.672 Å) (Data not shown).

Taken together, these results strongly suggest that these baseplate proteins containing CBMs are likely involved in binding to cell surface polysaccharides upon Vp4 and Deep-Blue adsorption to their hosts.

### Polysaccharide moieties are involved in the adsorption of Vp4 and Deep-Blue

**Fig 5:**
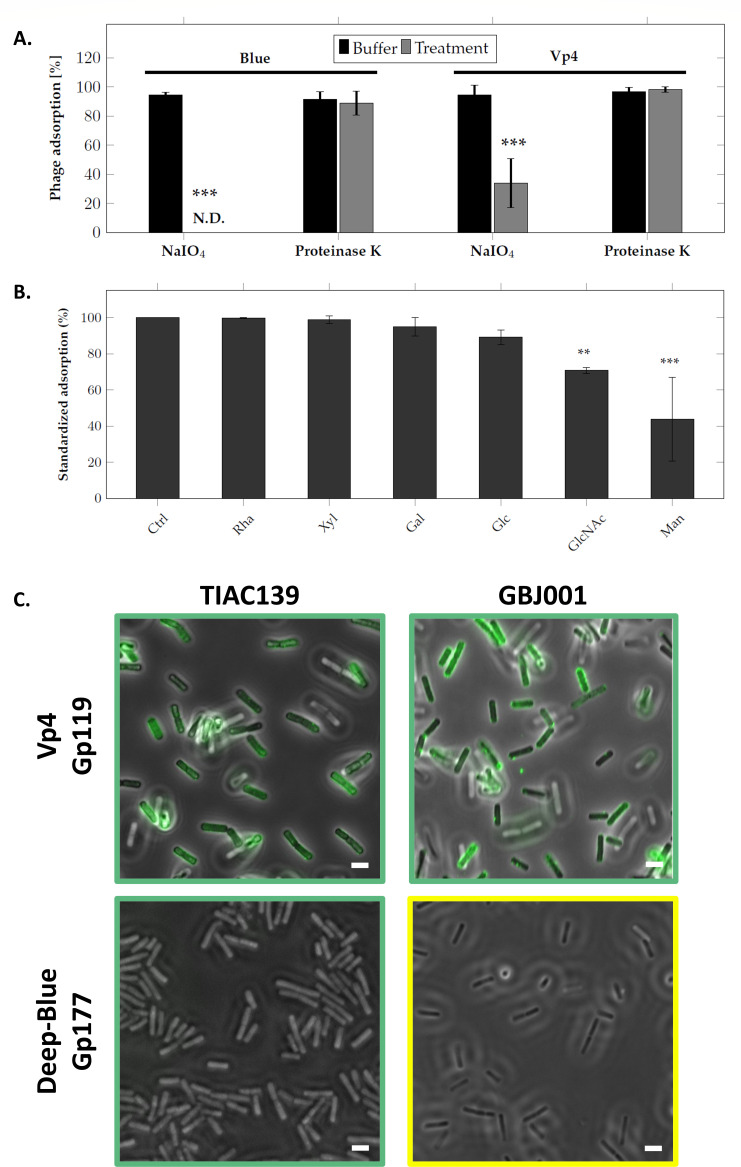
Implication of polysaccharide moieties in Deep-Blue and Vp4 adsorption to their hosts. (A) Assessment of the nature of Deep-Blue and Vp4 receptor. Deep-Blue and Vp4 hosts (*B. weihenstephanensis* Si0239 and *B. thuringiensis* GSX002, respectively) were treated with either sodium periodate, to alter carbohydrates, or proteinase K, to alter proteins before performing a phage adsorption assay. Statistical analyses are based on Tukey’s law, and asterisks indicate statistical differences (***P < 0.001). (B) Evaluation of saccharidic moiety involved in Vp4 adsorption. Competition assays were performed by incubating Vp4 with different sugars followed by an adsorption assay with its host strain (i.e., *B. thuringiensis* GSX002). Results are derived from three independent experiments and standardized with the adsorption of the control treatment that was set to 100% for each experiment. Statistical analyses are based on Tukey’s law, and asterisks indicate statistical differences (**P < 0.05, ***P < 0.001). (C) Cell wall decoration assay of Vp4-Gp119 and Deep-Blue-Gp177 to bacteria of the *B. cereus* group. Both proteins were fused to a GFP tag to assess their binding abilities. Exponentially growing *Bacillus* cells were incubated with ca. 50 µg of proteins and observed using an epifluorescence microscope. The tested strains are indicated on the top. The overlays between the contrast phase and fluorescent images are shown. The scales represent 5 µm. Green frames represent sensitive strains while yellow frames represent lysed strains. Ctrl, control (no sugar added); Gal, galactose; Glc, glucose; GlcNAc, N-acetyl glucosamine; Man, mannose; Rha, ramnose; Xyl, xylose.

Receptors recognized by phages can be proteins and/or carbohydrates located at the bacterial surface (11, 14). To test which type of receptors are recognized by Deep-Blue and Vp4, their bacterial hosts (i.e., *B. weihenstephanensis* Si0239 and *Bacillus thuringiensis* GSX002, for Deep-Blue and Vp4, respectively) were treated with periodate and proteinase K before phage adsorption assays (Fig. 5A). When the host surface proteins were damaged using proteinase K, no visible effect on Deep-Blue and Vp4 adsorption could be observed. Conversely, pretreatment with periodate, which alters carbohydrate structures, severely impaired the adsorption of Deep-Blue and, to a lesser extent, that of Vp4. These results indicate that both phages rely on carbohydrate-containing receptors, which is in agreement with the CBMs found in their baseplate proteins.

Cell surface carbohydrates assume various functions within the *B. cereus s.l*. group and have been previously shown to be widely distributed across the surface at every stage of their life cycle (i.e., from vegetative cells to spores) (58), which renders them suitable targets for phage binding upon adsorption. To further document which type of polysaccharide moieties might be involved in the adsorption process, competition assays incubating Vp4 phage with various sugars found in secondary cell wall polysaccharides were performed. As shown in Fig. 5B, mannose and N-acetyl glucosamine interfered with the adsorption of Vp4, which suggests the potential implication of these, or similar molecules, in the phage adsorption process.

As shown earlier, Vp4 Gp119 displays CBM folds, indicating a potential role for this BW3 in host binding, whereas its Deep-Blue homolog, Gp177, lacks such CBMs and is likely dedicated solely to structural function. To assess this hypothesis, both proteins were fused to an N-terminal GFP and tested in cell wall decoration assays. While Gp177 could not bind to any of the tested strains, Gp119 specifically decorated the surface of *B. cereus* sensitive strains, including the TIAC139 strain (Fig. 5C). Quite interestingly, this strain is the only sensitive strain that is not decorated by Vp4’s RBP Gp112. Remarkably too, the first CBM of Gp119 is identified as a CBM16 family according to the CAZy nomenclature (PDB ID: 2ZEY) (59), which was previously shown to interact with a mannose-like structure upon adsorption (60). Gp119 is therefore likely binding mannose-like structure upon Vp4 adsorption, which is further suggested by the interference observed when Vp4 is incubated with mannose before adsorption (Fig. 5B).

The binding propensity of the other TPs displaying CBMs depicted in Fig. 4 could not be tested as their fusion with GFP did not yield soluble proteins.

### Deep-Blue and Vp4 encode atypical cell-puncturing devices

In myophages, the central part of the baseplate is occupied by a cell-puncturing device responsible for membrane penetration and local degradation of PG upon phage adsorption (27) (Fig. 1B). This central hub is composed of a conserved protein forming a hexameric hub and serving as a starting point for the tail tube polymerization. In Deep-Blue and Vp4, this protein corresponds to Gp181 and Gp115, respectively.

Membrane-piercing proteins often harbor coiled-coil segments which are commonly found in central spikes of Gram-positive phages, including A511 Gp99 (27). As mentioned earlier, the Vp4 tail spike is formed by Gp111, in which domains (e.g., R2 pyocin membrane-piercing spike, PDB ID:4S37) similar to those found in membrane-piercing proteins as well as coiled-coil segments were detected. Moreover, as CBM folds are present in this protein (Fig. 4A), it is also likely involved in host recognition and binding. In Deep-Blue, these cell-puncturing and host-binding activities seem to be distributed between two proteins: Gp188, for the membrane-piercing protein, and Gp186, for the host-binding protein, that are homologs to the N-terminal and C-terminal regions of Vp4-Gp111, respectively (Fig. 4A).

**Fig 6:**
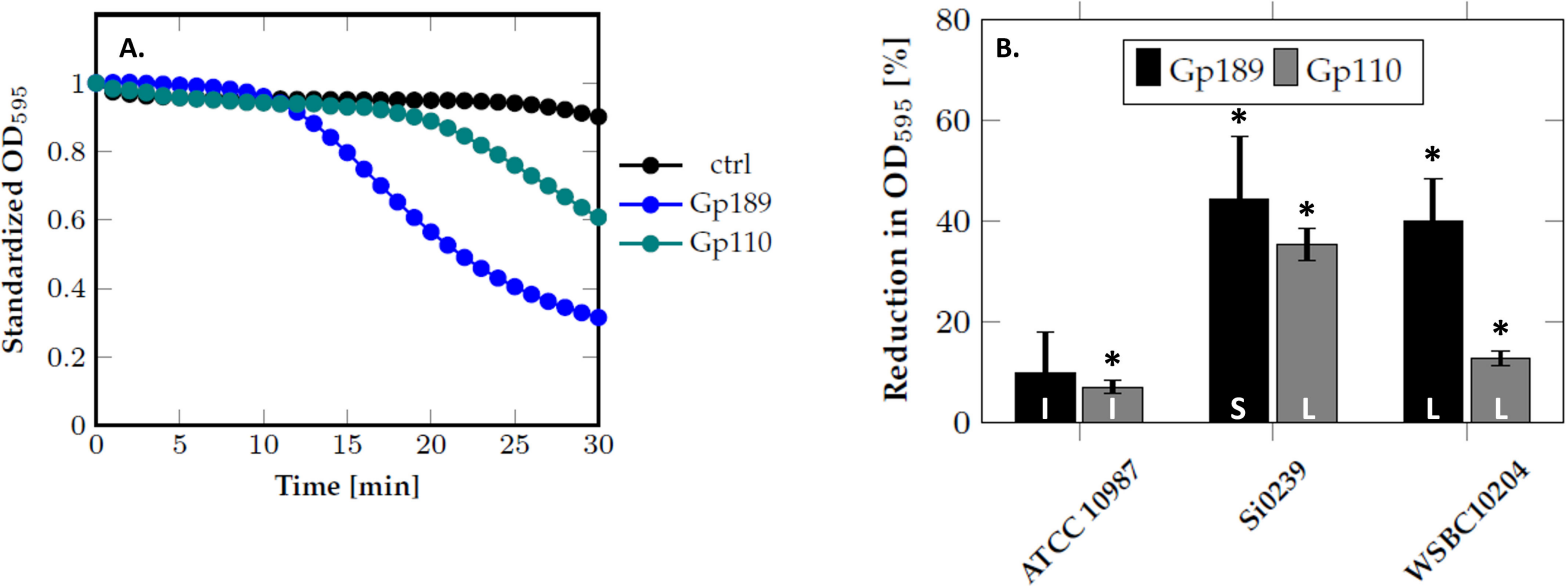
Bacteriolytic activity of the baseplate hub proteins (ectolysin) of phages Deep-Blue (Gp189) and Vp4 (Gp110). (A) Bacteriolytic activity of Deep-Blue Gp189 (blue) and Vp4 Gp110 (green) assessed by OD_595_ monitoring. The test was performed on exponentially growing *B. weihenstephanensis* Si0239 using 100 µg/mL of protein. “ctrl” indicates The control condition with the protein buffer. The data were standardized with respect to the OD_595_ at time 0. (B) The activity of Deep-Blue Gp189 (black) and Vp4 Gp110 (gray) was tested against several strains of the *B. cereus* group. The activity was assessed on exponentially growing cells using 100 µg/mL of proteins for 30 min. The results are expressed as a percentage of OD_595_ reduction compared to the control performed with the protein buffer. I, insensitive strain; L, lysed strain; S, sensitive strain. *Indicates lytic activity significantly different from 0 (*P < 0.05).

Deep-Blue Gp189 and Vp4 Gp110 share structural similarities in their N-termini with the phage A511 Gp98 central baseplate hub (BH), while they harbor a C-terminal peptidase domain from the NlpC/P60 family (pfam00877), absent in A511 Gp98. This supports their role as BH proteins involved in the local degradation of PG (Fig. 1A; Table 1). We produced these proteins and assessed their role as ectolysins in a turbidity reduction assay. Incubation of *B. weihenstephanensis* Si0239 with 100 µg/mL of Deep-Blue Gp189 or Vp4 Gp110 led to a decrease in the culture optical density (OD), confirming their protein lytic activity (Fig. 6A). As shown in Fig. 6B, this lytic activity is not limited to sensitive *B. cereus* strains.

## DISCUSSION

In this work, we performed a detailed analysis of the TPs that are part of Deep-Blue and Vp4 baseplates, two myophages belonging to the *Herelleviridae* family and infecting members of the *B. cereus* group. Both phages display almost identical gene order, the main differences being the presence of genes encoding a putative depolymerase (*gp104*) upstream of the *tmp* in Vp4 and an unknown TP (*gp187*) in Deep-Blue. The conserved baseplate components found in most contractile tail systems and forming the baseplate core (i.e., baseplate hub proteins BH1 and BH2) and wedges (i.e., baseplate wedge proteins BW1–BW3) were easily identified in Deep-Blue and Vp4 based on sequence identity and structural homologies. Our study also provides a structural analysis of the host adhesion devices used by these myophages to interact with their bacterial hosts, using AlphaFold2, which has previously been shown to be powerful for the study of siphophage tails (34–37).

Among the TPs, RBP, which is responsible for receptor recognition, is often the most variable component (61, 62). Deep-Blue and Vp4 encode Gp185 and Gp112, respectively, which were prime RBP candidates. First, no homolog was found among the TPs of *Listeria* phage A511, which is not surprising, given their host CW differences. For instance, wall teichoic acids, which are recognized by phages infecting *Listeria*, are not found in the CW of *B. cereus* strains (62, 63). Second, protein alignment between Deep-Blue Gp185 and Vp4 Gp112 revealed that their C-termini are less conserved than the rest of the proteins, which could explain, at least partially, the differences in host range of their corresponding phages. Finally, the structure predictions of Gp112 and Gp185 displayed typical characteristics of RBP structures. The C-terminal variable parts of Gp185 and Gp112 were fused to a GFP tag that showed their ability to bind to *B. cereus s.l*. strains, thereby confirming their implication in adsorption.

Interestingly, the binding spectra of Gp185 and Gp112 did not mirror the host range of their parental phages. In addition to sensitive strains, both proteins were also able to bind to strains that were only affected by lysis at high concentrations (i.e., no plaque formation). Similar observations were made for Gp108, the RBP of the *Listeria* phage A511, which also binds to strains onto which the phage was unable to form plaque (40). These wider binding ranges can have several potential explanations. The fact that many strains are only affected by lysis at high concentrations but are nonetheless recognized by Gp185 and Gp112 suggests that either intracellular resistance mechanisms prevent phage replication or that the attachment of Gp185 and Gp112 to their cognate receptor is not sufficient to initiate genome injection and subsequent steps of the phage life cycle. Therefore, one cannot exclude that other proteins are involved in the adsorption process as it is the case for other phages encoding several RBPs (64, 65).

In phage A511, a particular TP, Gp106, is also believed to be involved in adsorption (32). This protein forms pyramidal-shaped structures attached to the tail fibers, giving the baseplate a two-layer aspect (40). Upon attachment of A511 RBP (i.e., Gp108 which forms the distal part of the tail fiber), Gp106, which initially points upwards, reorients itself to interact with the CW, possibly via a binding domain formed by Gp107 (i.e., a putative chaperone). Protein alignment with A511 revealed that similar proteins are found in Deep-Blue (Gp176) and Vp4 (Gp120), and predictions of their structures retrieved CBM folds homologous to those present in A511 Gp106, suggesting a similar implication during adsorption.

Strikingly, CBM folds were also detected in several TPs encoded by Deep-Blue (BS-Gp186) and Vp4 (BS-Gp111 and BW-Gp119). Although CBM-containing proteins have been described in several siphophages infecting Gram-positive hosts (22), to the best of our knowledge, they have not been reported in myophages so far. In siphophages infecting lactic acid bacteria, CBMs are mostly found in TPs likely to facilitate and/or improve phage adsorption (66–68). Of particular interest, Gp119, the BW protein of Vp4 harboring CBM folds that are absent from its Deep-Blue homolog, has been shown to bind to the *B. cereus s.l*. surface, but its precise involvement in Vp4 attachment remains so far unknown. The presence of CBMs in different TPs, combined with the observation that altering carbohydrates led to a reduced adsorption of Deep-Blue and Vp4, indicates that host polysaccharides play an important role in the recognition and attachment of these myophages. The presence of these CBMs seems to be conserved among myophages infecting *B. cereus* and differs from previously described myophages (27, 29–32). This would suggest that myophages preying on *B. cereus* hosts have evolutionarily selected the insertion of CBM folds to target the numerous polysaccharide moieties of the *B. cereus* CW upon adsorption.

Finally, the proteins composing the central needle appeared to be unique in Deep-Blue and Vp4, suggesting that their puncturing device differed not only from Gram-negative contractile tail systems but also from that of phage A511 infecting *Listeria* (32, 40, 69). The enzymatic activity of Vp4-Gp110 and Deep-Blue-Gp189 was also confirmed, which further supports their role as ectolysin, locally degrading CW upon phage genome injection.

Overall, this work provides the first insights into the proteins involved in the adsorption of myophages infecting the *B. cereus* group. Further works are needed to decipher the whole adsorption process and the exact involvement of each protein, including accessory proteins containing CBMs.

## MATERIALS AND METHODS

### Bioinformatic tools

Bioinformatic analyses of phage tail proteins were done by genome comparison using EasyFig (70) using tBLASTx with the following parameters: minimum length of 30 bp and maximum *E* value of 0.001. Protein sequence analyses were performed using Interpro (71) and BLASTp (72).

### AF2 structure predictions

Structure predictions of TPs were made using a ColabFold notebook (v.1.5.5 monomer ColabFold and v.2.3.2 multimer ColabFold), available online and accessed in March 2024 (73, 74). All protein structures were first predicted as monomer. Due to memory limitations, sequences longer than ca. 800 residues were split, with respect to domain limitations. The predicted structures were then submitted to the Dali server to identify the closest structural homologs in the PDB (75). Once individual domains were identified, we performed additional Dali predictions using each domain structure as an input. Trimerization of Gp112 and Gp185 was performed using AF multimer ColabFold. Structure analysis and representation were performed using ChimeraX (76).

### Bacterial strains and growth conditions

Bacterial strains and plasmids used in this work can be found in Table 3. Bacteria were grown in lysogeny broth (LB) or LB-agar at 37°C for *E. coli* or 30°C for *Bacillus*. When required, media were supplemented with antibiotics (Sigma-Aldrich, Overijse, Belgium): kanamycin (selection for pET30 plasmid) and/or chloramphenicol [Rosetta (DE3)] at a final concentration of 50 µg/mL.

**TABLE 3:** Bacterial strains and plasmids used in gene cloning and expression experiments

Strain or plasmid	Species/purpose	Source
Strains		
10-beta	*E. coli*, cloning strain	NEB^*a*^
BL21(DE3)	*E. coli*, T7 expression strain	Merck^*b*^
Rosetta(DE3)	*E. coli*, T7 expression strain containing codons rarely used in *E. coli*	Merck^*b*^
Plasmids		
pET30a	Expression vector	NEB^*a*^
pUC18::*gfp*	GFP amplification	Clontech/Takara^*c*^
pET30::*gp*189	Test of Deep-Blue Gp189 lytic activity	This work
pET30::*gfp*(linker)::*gp*185_Cter	Test of Deep-Blue Gp185_Cter binding activity (aa 600–907)	This work
pET30::*gfp*(linker)::*gp*177	Test of Deep-Blue Gp177 binding activity	This work
pET30::*gp*110	Test of Vp4 Gp110 lytic activity	This work
pET30::*gfp*(linker)::*gp*112_Cter	Test of Vp4 Gp112_Cter binding activity (aa 589–896)	This work
pET30::*gfp*(linker)::*gp*119	Test of Vp4 Gp119 binding activity	This work

^*a*^ NEB, New England BioLabs, Ipswich, MA, USA.

^*b*^ Merck, Darmstadt, Germany.

^*c*^ Clontech/Takara, Saint-Germain-en-Laye, France.

### Phage host spectrum assessment

The phage host spectra were established using the spot-on-plate method. Briefly, 5 mL of soft agar (LB 0.5% agar, 3-mM CaCl_2_, and 3-mM MgSO_4_) was inoculated with 100 µL of exponentially growing cells (4 h) and poured on an LB-agar plate. After drying, 10-fold serial dilutions of phage suspensions were spotted, and the plate was subsequently incubated at 30°C overnight (O/N). Three types of phage sensitivities were distinguished: strains that are sensitive (presence of lysis plaques), insensitive (no lysis plaque or lysis area), or lysed (presence of lysis areas at high concentrations but no individual plaques).

### Bacterial cell wall treatments to assess the nature of phage receptor and influence of various sugars on Vp4 adsorption

To assess the nature of the phage receptors, 500 µL of an O/N culture of *B. weihenstephanensis* Si0239 (Deep-Blue preferred host) or *B. thuringiensis* GSX002 (Vp4 preferred host) was collected by centrifugation (10,000 × *g*, 10 min), washed with phosphate-buffered saline (PBS) (Sigma-Aldrich), and resuspended in 500 µL of either 100-mM NaIO_4_ (dilution buffer 50-mM CH_3_COONa, pH 5.2) or 0.2-mg/mL proteinase K (dilution buffer 20-mM Tris-HCl, 100-mM NaCl, pH 7.5). Control reactions used PBS and the respective dilution buffers. The NaIO_4_ treatments were incubated for 2 h at room temperature (RT) protected from light, and the proteinase K treatments were incubated for 1 h at 45°C. The cells were then centrifuged (10,000 × *g*, 10 min) and washed twice with PBS and resuspended in 950 µL of LB (supplemented with 3-mM CaCl_2_ and 3-mM MgSO_4_) and 50 µL of phage suspension (ca. 10^6^ PFU/mL). To evaluate the adsorption efficiency to the treated bacteria, phage/bacteria suspensions were incubated for 20 min at 30°C, then centrifuged (10,000 × *g*, 10 min, 4°C) to remove the bacteria and adsorbed phages. The supernatant was filtered on 0.45 µm (VWR, Oud-Heverlee, Belgium), and the remaining phage titer was evaluated by the double-layer agar assay in which serial dilutions of the phage suspension were mixed with their preferential host and poured as a top layer on agar plates (77).

The impact of various sugars on Vp4 adsorption was tested by incubation of 25 µL of Vp4 suspension (ca. 10^6^ PFU/mL) with 200 µL of 0.5 M of sugar solution (rhamnose, xylose, galactose, glucose, N-acetylglucosamine, and mannose) for 1 h at RT. GSX002 culture (275 µL of ca. 10^6^ CFU/mL) was then added to assess Vp4 adsorption as described above.

### Molecular cloning

PCR amplifications of target genes were performed using the primers listed in Table 4 and the Q5 High-Fidelity DNA polymerase [New England Biolabs (NEB), Leiden, The Netherlands]. The resulting amplicons and the expression vector, pET30a, were cleaved using appropriate restriction enzymes purchased from NEB and then ligated using the T4 DNA ligase (Promega, Madison, WI, USA). PCR and restriction products were purified using the GeneElute PCR Clean-Up Kit (Sigma-Aldrich). Plasmids were transformed into competent *E. coli* 10-beta cells, and transformants were identified by PCR. Plasmids were then extracted using the GeneElute Plasmid MiniPrep Kit (Sigma), verified by Sanger sequencing (Macrogen, Amsterdam-Zuidoost, The Netherlands), and subsequently transformed into the expression strain BL21 (DE3) or Rosetta (DE3) (only for pET30::*gfp::gp119*).

**TABLE 4:** Primers used in this work^*a*^

Target	Name	Sequence (5′ > 3’)
*gfp*	GFP_EcoRI_F	**TTCCGAATTC**AAAGGAGAAGAACTTTTCACTGGAG
	GFP_EagI_linker_R	**TTCGGCCGTCCACTACCTGATCCACTACC**TTTGTAGAGCTCATCCATGCC
*gp189*	gp189_BamHI_F	**TATGGATCC**ACAACGATTGTTAAACGCTATCC
	gp189_SalI_R	**TCTGTCGAC**TTATCCTGTAAACCTTCTTACGTG
*gp185_Cter*	gp185_Cter_EagI_F	**TACGGCCG**CAGTGGATTGCACAGGG
	gp185_XhoI_R	**TATACTCGAG**AAACATTGATTGAAATGTCATTGTC
gp177	gp177_EagI_F	**TTCGGCCG**TCATTTCTAAAACATCTACATCCTGGAT
	gp177_XhoI_R	**TATACTCGAG**CTATACTGTAAATTCAATCATGAATTGTTCGTC
*gp110*	gp110_NcoI_F	**ATACCATGGTT**GTACAGATTCAAAAGAGGTACC
	gp110_XhoI_R	**TATACTCGAG**TTAACCCGAATACCTTCTTACG
*gp112_Cter*	gp112_Cter_EagI_F	**TTCGGCCG**GGTATGGTAAATCTAAAAGCTGAC
	gp112_XhoI_R	**TATACTCGAG**CATTTGTCCTGTCTCCTATCTAA
*gp119*	gp119_EagI_F	**AACGGCCG**AGCTTTATGAAATATTTACATCCTC
	gp119_XhoI_R	**TATACTCGAG**TTATTTATTCTTGATATCGATAATAAACTCTTCG

^*a*^ Note that the primer extended ends are highlighted in bold.

In order to test the protein binding ability of the RBP candidates, only the regions corresponding to the variable C-termini of Deep-Blue Gp185 (residues 600–907) and Vp4 Gp112 (residues 589–896) were cloned in the expression vector and fused to an N-terminal *gfp* to yield pET30::*gfp::gp185_Cter* and pET30::*gfp::gp112_Cter*, respectively. For the other putative ancillary binding proteins (i.e., Deep-Blue Gp177 and Vp4 Gp119), the whole genes were fused to the *gfp* gene. For all GFP fusions, a linker composed of seven alternating glycine and serine residues was added between the GFP and the target proteins. The candidate ectolysin genes (i.e., Deep-Blue *gp189* and Vp4 *gp110*) were cloned in pET30a in order to test the lytic activity of their corresponding proteins.

### Protein expression and purification

O/N cultures of the expression strain *E. coli* BL21 (DE3) or Rosetta (DE3) containing the recombinant vectors were sub-cultured in LB broth (dilution 1:20) and incubated at 37°C, 180 rpm, until the optical density (OD_600_) reached 0.5–0.8, and induced with 0.5 or 1.0-mM isopropyl β-D-1-thiogalactopyranoside (IPTG) (Sigma-Aldrich). Cultures were incubated at 180 rpm during 6 h at 28°C or 24 h at 22°C to allow protein expression. The bacterial pellet was then collected by centrifugation (4,000 × *g*, 4°C, 15 min) and stored at −20°C. For purification, the bacterial pellet was thawed on ice for 30 min and resuspended in lysis buffers NPI10 (50-mM NaH_2_PO_4_, 300-mM NaCl, 10-mM imidazole, 1-mg/mL lysozyme, pH 8) or Tris10 (25-mM Tris-HCl, 100-mM KCl, 10-mM imidazole, 1-mg/mL lysozyme, pH 8) supplemented with a protease inhibitor cocktail (1× SIGMAFAST Inhibitor Cocktail Tablet, EDTA-Free; Sigma-Aldrich) and incubated for 2 h on ice to lyse the cells. Three units/mL of Benzonase Nuclease (Sigma-Aldrich) was added to reduce viscosity (15 min of incubation). Soluble proteins were recovered in the supernatant by centrifugation at 10,000 × *g* for 30 min at 4°C, which was filtered on 0.45 µm. The recombinant proteins were then purified using a Ni-NTA column (Qiagen, Hilden, Germany) according to the manufacturer’s recommendations. The purity was assessed by SDS-Page (Bio-Rad, Hercules, CA, USA), and the protein concentration was determined by Bradford assay (78).

### Cell wall decoration assay

A cell wall decoration assay was used to assess the binding of the GFP-fused proteins to the bacterial cells (79). Briefly, 300 µL of exponentially growing cells (4 h) was collected by centrifugation (10,000 × *g*, 5 min), washed twice with sodium-magnesium (SM) buffer (50-mM Tris-HCl, 100-mM NaCl, 8-mM MgSO_4_, pH 7.5), and resuspended in 100 µL of SM buffer. Twenty to fifty micrograms of purified GFP fused proteins was added to the bacterial suspension and incubated for 20 min at RT. Control reactions used the protein buffer and purified GFP. The bacterial suspensions were then centrifuged (10,000 × *g*, 5 min, 4°C) and washed twice with cold SM buffer. Bacteria were finally observed under an epifluorescent microscope (Leica AF600) using a filter with an excitation wavelength ranging from 460 to 500 nm and an exposure time of 300 ms. Images were obtained using the Leica LAS AF software.

### Turbidity reduction assay

The lytic activity of the two potential ectolysins was evaluated using a turbidity reduction assay (80) which consists in measuring the decrease of the OD_595_ when *B. cereus* strains are subjected to Gp189 or Gp110. Briefly, exponentially growing bacterial cultures (4 h) were centrifuged (10,000 × *g*, 5 min) and resuspended in either 25-mM (Gp189) or 50-mM (Gp110) Tris-HCl, pH 8, and the OD_595_ was adjusted between 0.8 and 1.0. The bacterial suspension was then transferred to each well of a 96-well plate and mixed with 100 µg/mL of ectolysins. Control wells were realized using protein buffers. The plate was incubated at 37°C (Gp189) or 30°C (Gp110) in a Multiskan FC Microplate spectrophotometer (Thermo Fisher Scientific, Watham, MA, USA) where the OD_595_ was monitored every minute for 30 min.

## Data Availability

Phage genomes and tail protein sequences are available at National Center for Biotechnology Information GenBank with the following accession numbers: Deep-Blue, NC_031056; Vp4 tail module, PP554270–PP554291; BC01, MH487649.1; BCU4, JN797798.1; Kioshi, MH638312.1; Anthony, MF498901.1; Spock, KF669662.1; Troll, KF208639.2.

## SUPPLEMENTAL MATERIAL

Supplemental material jvi.00745-24-s0001.docx Fig. S1 and S2; Tables S1 and S2.

ASM does not own the copyrights to Supplemental Material that may be linked to, or accessed through, an article. The authors have granted ASM a non-exclusive, world-wide license to publish the Supplemental Material files. Please contact the corresponding author directly for reuse.
