# Supplementary material for: Deciphering the adsorption machinery of Deep-Blue and Vp4, two myophages targeting members of the Bacillus cereus group
Source: J Virol. 2024 Aug 23;98(9):e00745-24. doi: 10.1128/jvi.00745-24 (PMC11406892; doi:10.1128/jvi.00745-24)
Supplement: Supplemental material — Fig. S1 and S2; Tables S1 and S2. [file jvi.00745-24-s0001.docx]

**
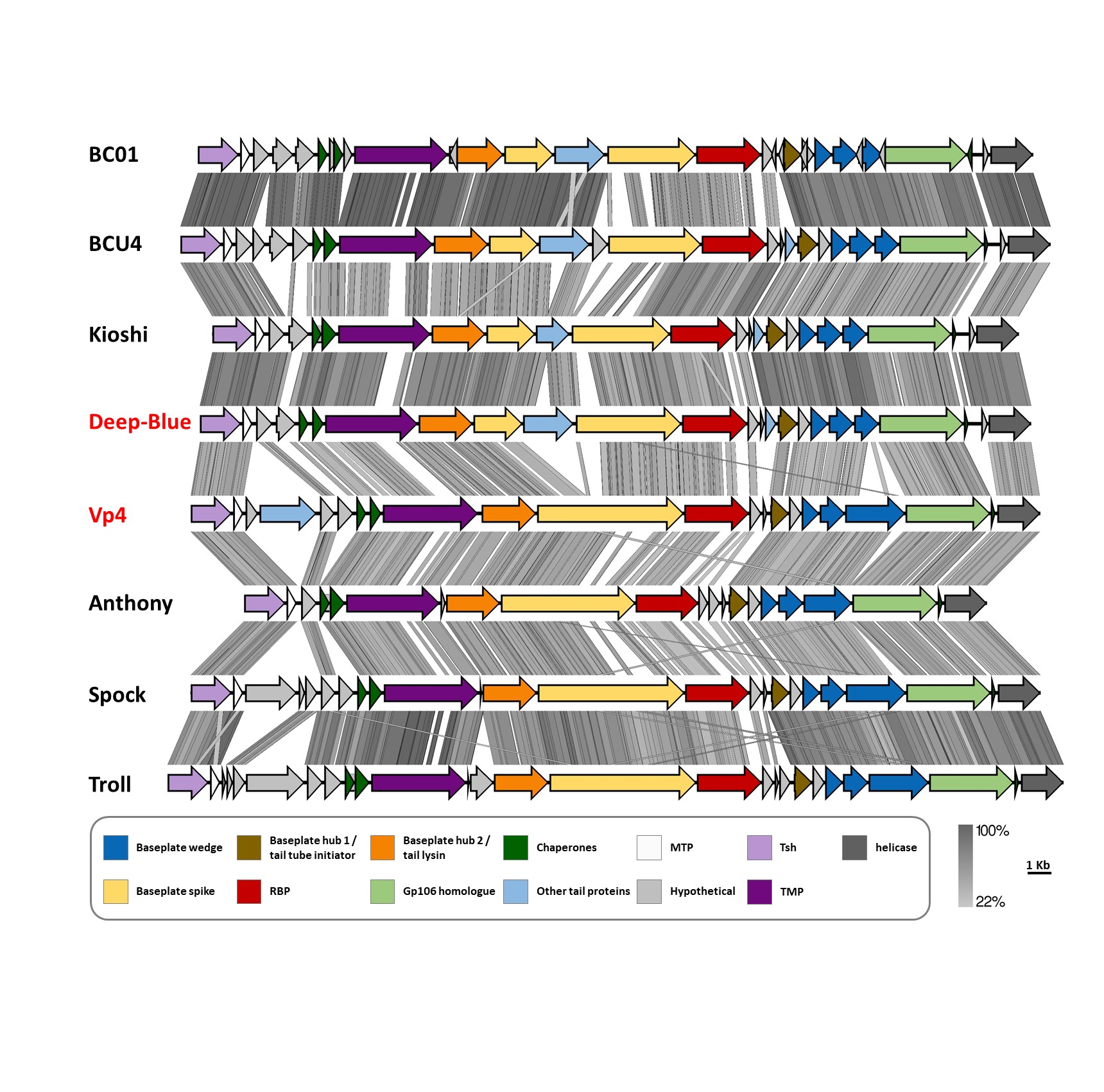
**

**Figure S1 - Comparison of Deep-Blue and Vp4 tail encoding regions with those of other myoviruses infecting members of the *B. cereus* group.** The alignment was generated with EasyFig using tBLASTx and the gradient (gray) scale of gene identity is indicated on the right together with the scale of the genetic loci (in kb). The color coding is indicated at the bottom. BH: Baseplate Hubs; BS: Baseplate Spike; BW: Baseplate Wedges; MTP: Major Tail tube Protein; RBP: Receptor Binding Protein; TMP: Tape Measure Protein; Tsh: Tail sheath.


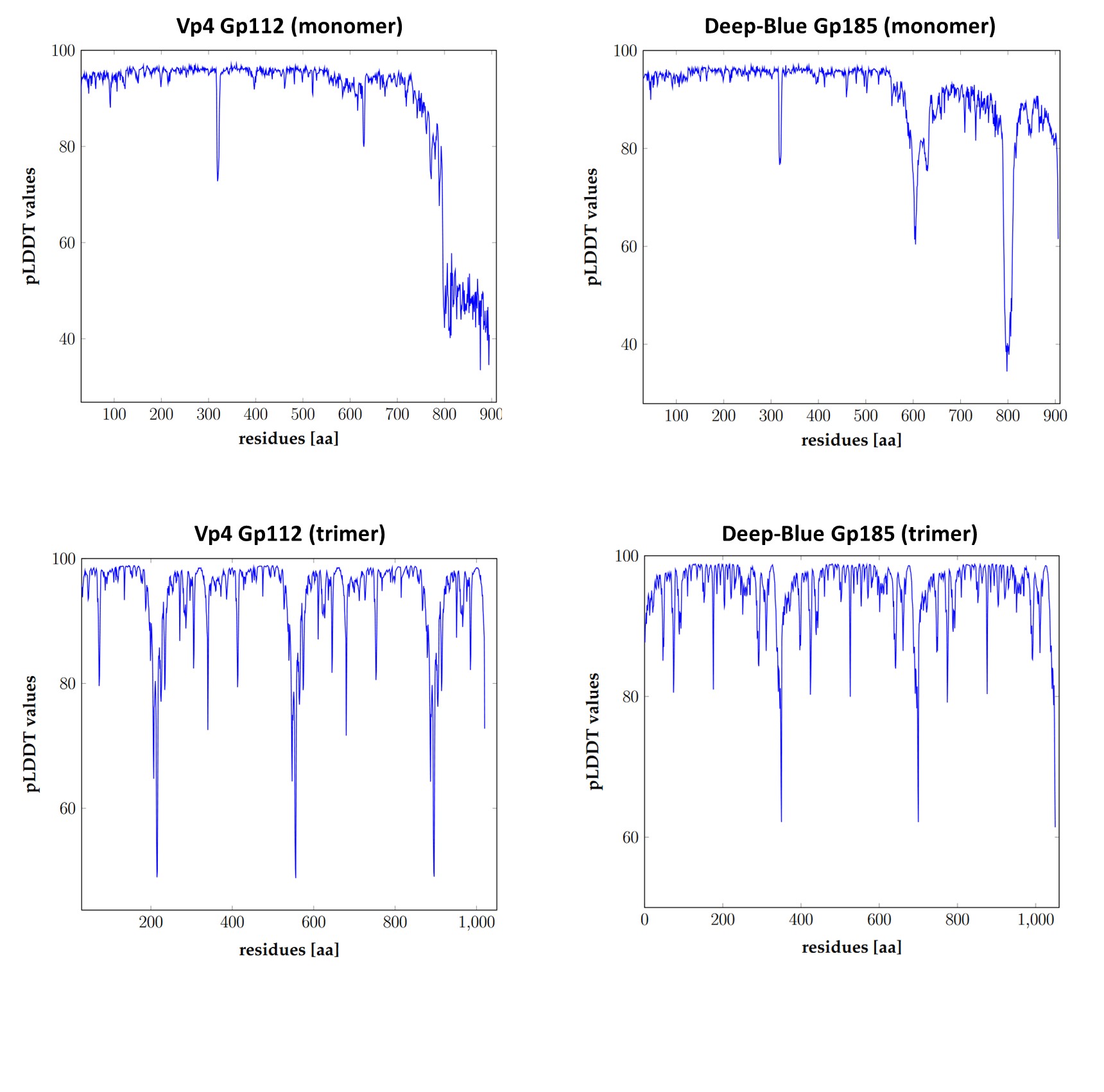


**Figure S2 - pLDDT value for Vp4 Gp112 and Deep-Blue Gp185 (monomeric and trimeric structures).** The pLDDT values for each residue along protein sequences are plotted.

**Table S1 – Putative functions of the tail proteins found in Deep-Blue and Vp4 phages and their similarities to phage A511 proteins.** The numbers found in the column “Identity” indicate the percentage of protein identity between the related proteins in Deep-Blue and Vp4. “111_N” and “111_C” represent the N- and C-terminus of the Vp4 protein Gp111, respectively. “-“ indicates that the protein is absent of the tail region of the corresponding phage.

| **Putative function** | **Deep-Blue** | | **Vp4** | | **Identity (%)** | **A511** | |
| --- | --- | --- | --- | --- | --- | --- | --- |
|  | **Protein (Gp #)** | **Size (aa)** | **Protein (Gp #)** | **Size (aa)** |  | **Protein (Gp #)** | **Size (aa)** |
| Tail sheath protein | **197** | 569 | **101** | 569 | 65 | **93** | 562 |
| Tail tube protein | **196** | 142 | **102** | 142 | 87 | **94** | 140 |
| Hypothetical protein | **-** | - | **103** | 172 | - | **-** | - |
| Tail depolymerase | **-** | - | **104** | 790 | - | **-** | - |
| 3D containing protein | **195** | 227 | **105** | 204 | 54 | **-** | - |
| Hypothetical protein | **194** | 278 | **106** | 225 | 33 | **-** | - |
| Tail assembly chaperone | **193** | 139 | **107** | 139 | 63 | **95** | 147 |
| Tail assembly chaperone | **192** | 158 | **108** | 162 | 57 | **96** | 197 |
| Tape measure protein | **191** | 1,281 | **109** | 1,316 | 48 | **97** | 1,242 |
| Baseplate hub 2/Tail lysin | **189** | 746 | **110** | 756 | 67 | **98** | 795 |
| Central spike/needle | **188** | 679 | **111_N** | 2,033 | 55 | **99** | 510 |
| Tail protein | **187** | 701 | **-** | - | - | **-** | - |
| Central spike/needle | **186** | 1,463 | **111_C** | 2,033 | 63 | **-** | - |
| RBP | **185** | 906 | **112** | 895 | 49 | **-** | - |
| Hypothetical protein | **184** | 163 | **113** | 171 | 41 | **-** | - |
| Hypothetical protein | **183** | 62 | **114** | 65 | 35 | **-** | - |
| Cysteine peptidase | **182** | 163 | **-** | - | - | **-** | - |
| Baseplate hub 1 | **181** | 275 | **115** | 257 | 58 | **100** | 237 |
| Hypothetical protein | **180** | 174 | **116** | 175 | 60 | **101** | 177 |
| Baseplate wedge 1 | **179** | 249 | **117** | 246 | 60 | **102** | 236 |
| Baseplate wedge 2 | **178** | 348 | **118** | 348 | 66 | **103** | 348 |
| Baseplate wedge | **-** | - | **-** | - | - | **104** | 1,309 |
| Baseplate wedge 3 | **177** | 341 | **119** | 831 | 52 | **105** | 173 |
| Tail fiber/VrlC | **176** | 1,169 | **120** | 1,174 | 69 | **106** | 1,151 |
| Chaperone | **175** | 63 | **121** | 64 | 46 | **107** | 73 |
| Hypothetical protein | **174** | 94 | **-** | - | - | **-** | - |
| RBP | **-** | - | **-** | - | - | **108** | 430 |
| Chaperone | **-** | - | **-** | - | - | **109** | 136 |

**Table S2 - Tail proteins of Deep-Blue and Vp4 and their homologues in other contractile tail systems.** The protein identification numbers of each homologue are indicated for the *Escherichia coli* myoviruses Mu, P2 and T4, the R-type pyocin from *Pseudomonas aeruginosa* and the contractile injection systems of the antifeeding prophage (AFP) from *Serratia entomophila* and the *Photorhabdus* virulence cassette (PVC). Abb.: Abbreviations.

| **Tail proteins** | **Abb.** | **Deep-Blue** | **Vp4** | **Mu** | **P2** | **T4** | **R-type pyocin** | **AFP** | **PVC** |
| --- | --- | --- | --- | --- | --- | --- | --- | --- | --- |
| **Tail sheath** | Tsh | 197 | 101 | 39 | FI | 18 | PA0622 | Afp2/3/4 | Pvc2/3/4 |
| **Tail tube** | MTP | 196 | 102 | 40 | FII | 19 | PA0623 | Afp1 | Pvc1 |
| **TMP** | TMP | 191 | 109 | 42 | FT | 29 | PA0625 | Afp14 | Pvc14 |
| **Central hub complex** | BH1 | 181 | 115 | 43 | U | 48/54 | PA0623 | Afp5 | Pvc5 |
|  | BH2 | 189 | 110 | 44 | D | 27 | PA0628 | Afp8 | Pvc8 |
| **Baseplate spike** | BS | 188 | 111 | 45 | V | 5 | PA0616 | Afp8 | Pvc8 |
| **Baseplate wedge** | BW1 | 179 | 117 | 46 | W | 25 | PA0617 | Afp9 | Pvc9 |
|  | BW2 | 178 | 118 | 47 | J | 6 | PA0618 | Afp11 | Pvc11 |
|  | BW3 | 177 | 119 | 48 | I | 6/7 | PA0618  PA0619 | Afp11 Afp12 | Pvc11 Pvc12 |
| **RBP** | RBP | 185 | 112 | 49 | H | 12 | PA0620 | Afp13 | Pvc13 |
